# Age-Based Comparative Analysis of Colorectal Cancer Colonoscopy Screening Findings

**DOI:** 10.3390/medicina59112017

**Published:** 2023-11-16

**Authors:** Ilona Vilkoite, Ivars Tolmanis, Hosams Abu Meri, Inese Polaka, Linda Mezmale, Aivars Lejnieks

**Affiliations:** 1 Health Centre 4, LV-1012 Riga, Latvia; 2 Digestive Diseases Center GASTRO, LV-1079 Riga, Latvia; 3 Department of Doctoral Studies, Riga Stradins University, LV-1007 Riga, Latvia; 4 Department of Internal Diseases, Riga Stradins University, LV-1007 Riga, Latvia; lejnieks@latnet.lv; 5 Institute of Clinical and Preventive Medicine, University of Latvia, LV-1586 Riga, Latvia; inese.polaka@lu.lv (I.P.); linda.mezmale@lu.lv (L.M.); 6 Riga East University Hospital, LV-1038 Riga, Latvia

**Keywords:** colorectal cancer, colorectal polyp, colorectal adenoma, young adults, adenoma detection, early onset colorectal cancer, CRC

## Abstract

*Background and Objectives*: Colorectal cancer (CRC) incidence is rapidly emerging among individuals <50 years, termed as early-onset colorectal cancer (EOCRC). This study aimed to probe variations in tumorigenic pathology and relevant manifestations (polyp and adenoma incidence) between suspected cases of EOCRC and late-onset CRC (LOCRC; ≥50 years of age). *Materials and Methods*: Between September 2022 and February 2023, colonoscopy-based screening data from 1653 patients were included in this study. All eligible participants were divided into two groups, depending upon patient age, where Group 1 consisted of 1021 patients aged <50 years while Group 2 consisted of 632 patients aged ≥ 50 years. Polyp samples were collected when identified peri-procedurally and characterized according to World Health Organization criteria. *Results*: Polyp detection rate was 42% for the <50-year age group, while this was 76% for the ≥50-year age group. Additionally, the <50-year age group predominated in hyperplastic polyp manifestation, particularly within the rectum and sigmoid colon. In addition, the ≥50-year age group had increased prevalence of serrated polyps and differing adenoma manifestations. *Conclusions*: This investigation served to highlight the importance of age stratification for CRC colonoscopy-based screening effectiveness, with particular reference to evaluations that are based on polyp localization within differing colon regions.

## 1. Introduction

Colorectal cancer (CRC) is the third most prevalent cancer type globally [1,2]. Epidemiological studies suggested that genetic and lifestyle factors such as smoking, alcohol intake, obesity, and reduced physical activity all increase the risk for CRC development [3]. Considering CRC incidence rate is increasing steadily on a global scale, particularly among individuals under the age of 50 years [4]. Such a clinical emergence has recently been coined as early-onset colorectal cancer (EOCRC) and has a deep genetic-based pathogenesis for approximately 80% of cases, typically developing within the distal segments of the colon and the rectum, and mainly affecting noncolored individuals [5]. In addition, most EOCRC tumors were found to have hypomethylations in tandem with chromosomal/microsatellite instability [5].

The pathogenesis of colorectal polyps, that can potentially transform into colon tumor tissue, has multiple etiologies, including inflammation and autophagy pathways, and typically involving oxidative stress, DNA injury, together with epigenetic dysfunction [6]. Autophagy is of importance in CRC development as it was found to contribute key genes, such as Beclin 1 and microtubule-associated protein 1 light chain 3 alpha (LC3) and autophagy-related gene 5 (ATG5) [6]. Such genes—through the promotion of active surveillance pathways—could possibly trigger CRC pathogenicity within the gastrointestinal tract [6]. Other findings linked to CRC pathogenesis include the specific presence of agrin biomarker within the muscularis mucosa of sessile serrated lesions (carrying highest malignancy development risks), unlike for hyperplastic polyps (having low malignancy risks), following immunohistochemistry assessments [7]. Apart from agrin, differential gene expression analyses and immunohistochemical assays also recognized serine peptidase inhibitor (SERPINE2) and TIMP metallopeptidase inhibitor 1 (TIMP1) to be upregulated within sessile serrated lesions and hyperplastic polyps, though had reduced presence within tubular adenomas and not present at all within healthy colon tissue [7]. One separate study also found extracellular nicotinamide phosphoribosyltransferase (eNAMPT) to have prominent malignant potential for colon polyp pathogenesis and development [8], where eNAMPT is considered a cancer metabokine [8].

Interestingly, one particular study conducted in Turkey evaluated 271 CRC patients presenting at their oncology clinic, whereby 100 patients from this cohort were found to be EOCRC, albeit there were no clinicopathological variations across EORC or late-onset CRC (LOCRC) patients in this specific study [9]. Additionally, the recent study conducted earlier in 2023 by Huntley and colleagues aimed at performing a modelling analysis for polygenic risk scores from UK cancer screening protocols [10]. This particular study employed age-stratified UK-based cancer incidence rates and determined polygenic risk scores for eight of the major tumor models, including CRC, within risk cohort quintiles (20%/quintile) [10]. In this study, the high-risk quintile for CRC was determined at 50–59 years of age [10]. Similarly, a separate study—also carried out earlier in 2023 by Zaborowski and colleagues—specifically focused on identifying pathogenic characteristics and endpoint outcome analyses within EOCRC patient cohorts [11]. This led to an international research effort, in conjunction with research in the early age colorectal cancer trends (REACCT) collaborative, whereby an overall cohort of 3378 CRC cases was analyzed in this study [11]. The results of this landmark and recent large-scale investigation found that the median age for EOCRC onset was 43 years (ranging between 18 and 49 years), with a marginal predominance in incidence rates within males (54.3%), and with 33% of such patients also having a family history of CRC [11]. Moreover, other findings identified within this seminal study were that 70.1% of tumors were located distal to the descending colon, and with 40% being also node-positive, while 20% of EOCRC cases also having microsatellite instabilities [11]. Another highly informative study was conducted by Deng and colleagues earlier in 2023, which focused on developing noninvasive predictive models for EOCRC, based mainly upon lifestyle analyses and other identified risk factors [12]. The results of this particular study, conducted over 811 EOCRC patients and 945 healthy controls, concluded that the consumption of sweet/fried foods items at a frequency of over thrice-weekly, was highly linked to EOCRC manifestation [12].

Consequently, following the monitoring of emerging incidence rates for EOCRC, guidelines for CRC screening are currently shifting towards the commencement of CRC screening at 45 years of age, rather than at 50 years, in order to increase diagnosis potential and minimize patient mortality risk from CRC [5].

Colonoscopy examination for colorectal adenoma detection, together with appropriate polypectomy, is still considered as the gold standard clinical diagnostic protocol for reducing CRC morbidity and mortality [13,14]. CRC was also found to develop primarily through the adenoma–carcinoma pathway, deriving from benign colonic adenomatous polyps, considered as precursor lesions of CRC [15]. Such pathological shifts within colon mucosa take place due to the influence of several factors, including genetic and epigenetic processes that result in the silencing of multiple tumor suppressor genes, activation of oncogenes, and the development of chromosomal instability [16,17]. CRC is also considered to be a suitable cancer model for screening, as most polyps detected during colonoscopy (including adenomas and sessile serous lesions) can be successfully removed endoscopically, thus providing CRC prophylaxis [18]. Consequently, the timely detection and removal of such polyps by polypectomy can prevent patients from developing CRC [19].

It is also well established that for every 1.0% increase in adenoma detection rate (ADR), the risk of interval CRC can be reduced by up to 3.0% [20]. In recent years, it has been observed that the prevalence and mortality for LOCRC has decreased, while—conversely—EOCRC incidence rates are on the rise [21,22]. In line with this highly concerning trend, Bailey et al. predicted that by 2030, the prevalence of colon and rectal cancers will increase by 90% and 124%, respectively, within individuals aged 20–34 years, and by 28–46%, respectively, within individuals aged 35–49 years [23]. Results from previous studies indicated that EOCRC progression at the time of diagnosis is more actively progressive, in comparison to counterpart LOCRC patients at the time of diagnosis, while prognosis is also often poor in EOCRC cases [24]. Consequently, colorectal neoplasia (CRC precursors) and EOCRC are of crucial clinical concern. Unfortunately, studies focusing specifically on young individuals in the context of CRC are currently limited and remain unclear.

STUDY AIM/S: This study aimed to probe histopathological variations in polyp and adenoma incidence profiles between <50 and ≥50-year-old patients, in an effort to provide further clinical and epidemiological feedback on CRC pathogenicity and incidence rates across varying human demographics. This is particularly relevant and novel as a research effort, since recent trends are highlighting the ongoing rise in EOCRC, whereby the majority of such patients are typically diagnosed with advanced-stage condition, due to lack of previous screenings (due to young age) [25]. The outcome of this study aimed to provide further awareness to healthcare professionals currently managing novel suspected CRC cases, according to age bracket and polyp location within the colon.

## 2. Materials and Methods

### 2.1. Study Population

This was single-center study conducted within our medical center. Overall, colonoscopy data from 1653 patients (665 males and 988 females) were included in this study. Such procedures were performed during the timeframe of September 2022–February 2023. Inclusion criteria were a minimum age of 18 years, together with availability of a signed informed consent form. Exclusion criteria were (a) a history of colonoscopy procedures; (b) inflammatory bowel diseases; (c) hereditary polyposis syndrome; (d) established CRC; (e) a history of colorectal surgery procedures; (f) contraindications for polypectomy; (g) incorrect bowel preparation on a Boston Bowel Preparation scale (BBPS) of 0–1 in any of the three bowel segments; (h) patients with standard contraindications to colonoscopy (including acute diverticulitis/suspected perforation); (i) incomplete colonoscopy procedure (technical difficulties/poor bowel preparation). All eligible patients were segregated into two groups, depending on patient age. Group 1 consisted of 1021 patients aged <50 years (one day prior to 50th birthday and younger), while Group 2 consisted of 632 patients aged ≥50 years (one day following 50th birthday and older).

This study was performed in accordance with the ethical guidelines of the Declaration of Helsinki 1975 and approved by the Central Medical Ethics Committee No. 01-29.1.2/1751.

### 2.2. Experimental Protocol

Colonoscopy examinations were performed through the Olympus™ EVIS V1^®^ video endoscopy platform together with ENDO-AID™ CADe^®^ artificial intelligence. All examinations were performed by two endoscopists, with overall experience of 23 years and performing approximately 2000 colonoscopy examinations annually. In addition, colonoscopy procedures were performed under anesthesiologist supervision using short-term intravenous propofol-based local anesthesia.

### 2.3. Colonoscopy Procedure

Bowel cleanliness was assessed through the BPPS scale. The timing for procedural instrument evacuation from cecum was not less than seven minutes. The colonoscopy procedure commenced by inspecting the anal canal and the rectum, performing an examination in retroversion to evaluate mucosal regions above the Z-line, and evaluating the condition of internal hemorrhoidal nodes. Once the anal canal mucosa was viewed in retroversion, the endoscope was advanced proximally into the sigmoid, descending, transverse, and ascending colon, until the cecum dome was reached. By moving the endoscope proximally into the colon, residual contents were evacuated from the colon. A mandatory requirement for a high-quality colonoscopy examination was image-based documentation of the appendix opening. When technically possible, a retroversion examination was also performed within the cecum. The right side of the colon was examined twice, returning distally from the cecum to the transverse colon, inspecting the mucosa within the narrow-band imaging (NBI) mode, and again proximal to the cecum. In order to detect flat polyps of the right colon, post-instrument evacuation from the cecum on the second occasion, mucosal staining with methylene blue solution was performed. Further inspection of colon mucosa was performed by gradually evacuating the endoscope and also carefully inspecting mucosal regions behind the folds.

Any detected polyp was described in the colonoscopy report according to the Paris [26] and NBI (narrow-band imaging) International Colorectal Endoscopic (NICE) [27] classifications, with polyp location within the colon (together with dimensions) being specified. Individual biopsied/ablated polyps were referred for morphological analyses. Whenever a polyp was identified, a minimum of two segments from the lesion were collected prior to polypectomy. Typically, colonic polyps were detected during instrument evacuation from the cecum, though if a polyp was detected in the direction towards the cecum, it was removed immediately. Due to patient data security concerns, endoscopic images were obtained solely through Olympus™.

### 2.4. Morphological Analyses

All collected lesion samples were analyzed and characterized according to World Health Organization (WHO) criteria, depending upon morphological characteristics [28]. Individual samples were described as follows: (a) serrated polyp/lesion (including hyperplastic polyp, sessile serrated lesion (SSL), SSL with dysplasia (SSL-D), traditional serrated adenoma (TSA), non-classified serrated adenoma); (b) low-grade dysplasia (LGD); (c) high-grade dysplasia (HGD); (d) superficial submucosal invasive carcinoma (SM-s; <1000 μm submucosal invasion); (e) deep submucosal invasive carcinoma (SM-d; >1000 μm submucosal invasion).

In addition, such samples were also stratified according to physical diameter dimensions, as follows: (a) diminutive (1–5 mm); (b) small (6–9 mm); (c) advanced (>10 mm).

### 2.5. Statistical Analyses

Concerning patient baseline sociodemographic/clinical characteristics and colonoscopy quality parameters, chi-square test was employed for categorical variables. A *p*-value of <0.05 was deemed to confirm statistical significance. Statistical analysis was performed using IBM™ SPSS^®^ (version 20) [29] and Fisher’s exact test.

## 3. Results

### 3.1. Patient Demographic Profiling

Overall, 1731 colonoscopy examinations were performed on 1653 patients participating in this study, with the remaining 78 patients excluded due to exclusion criteria implementation. Patient age approximated <50 years (Table A1 in Appendix A), with gender balance remaining across both study groups.

### 3.2. Colonoscopy Findings

**Figure 1:**
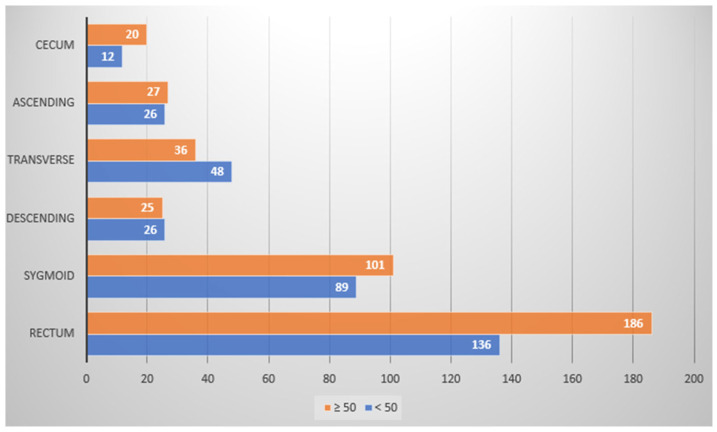
Patient populations (X-axis) with polyps detected during colonoscopy, according to colon regional location (Y-axis), and according to age bracket (above/50 years old (orange) and below 50 years old (blue)). See Table A2 in Appendix A for further details.

**Figure 2:**
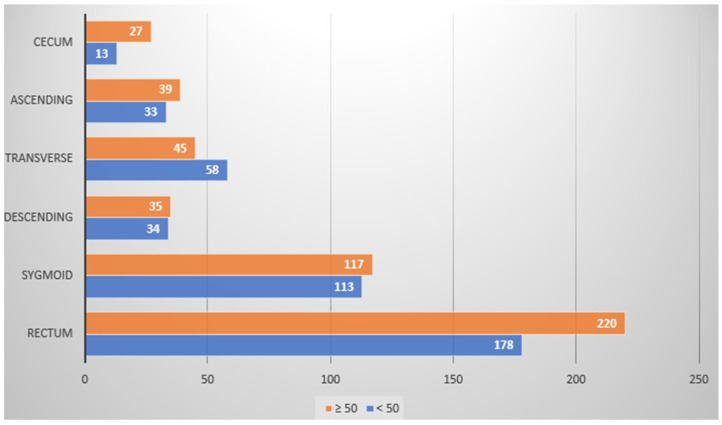
Polyp detection rate (X-axis) according to colon localization (Y-axis), and according to age bracket (above/50 years old (orange) and below 50 years old (blue)). See Table A3 in Appendix A for further details.

Overall, 732 (44.3%) among 1653 patients participating in this study were found to harbor a total of 912 colon polyps (polyp detection rate (PDR) being 55.2%), with patients ≥50 years having a nearly twofold increase in polyp incidence rate in comparison to the <50-year-old patient group (76% vs. 42% PDRs, respectively). Most polyps were found across both patient groups within the rectal region, followed by the sigmoid region, though polyps were rarely identified within the cecum region (Table A2 and Table A3 in Appendix A; Figure 1 and Figure 2). Patients with multiple polyps were also identified (Table A4 in Appendix A).

Polyp classification according to diameter dimension also identified 181 polyps having a diameter of >10 mm (Table A5 in Appendix A). In addition, polyp classification according to diagnosed pathology revealed that most polyps were prevalent within both study groups as hyperplastic polyps, predominantly manifesting within the rectal area (Table A6 in Appendix A). The largest number of hyperplastic polyps in both groups was detected in the rectum. It should be noted that in patients <50 years, the incidence rate of hyperplastic polyps was significantly higher in comparison to patients ≥50, identified within the rectum (Table A7 in Appendix A).

Serrated polyps were prevalent in 36 patients across both study groups, with the majority of such polyps being detected more frequently within the ≥50-year-old study group, particularly within the ascending colon and cecum (Table A8 and Table A9 in Appendix A). Similarly, LGD adenomas were prevalent mainly within rectal region for 285 patients, with this population mainly consisting of individuals ≥50 years of age (Table A10 and Table A11 in Appendix A). Finally, HGD adenomas were prevalent mainly within the rectal region for 158 patients, with this population mainly consisting of individuals ≥50 years of age (Table A12 in Appendix A).

No TSA, SSL-D, or unclassified serrated adenomas were identified pathologies across all investigated polyp samples throughout the course of this study. Table 1 below provides an overview of the study results, while detailed findings are illustrated in Appendix A.

## 4. Discussion

The recent decrease in the frequency of colorectal cancer, as reported, corresponds to the emergence and greater utilization of screening methods [30]. Considering the recognized pattern of colorectal cancer development, where progression from adenoma to cancer requires a minimum of 10 years, it is recommended to commence screening mid-risk patients at the age of 50 [31]. Guidelines concerning the detection of adenomas and quality standards in colonoscopy are established depending on the published literature, which demonstrates that 25% of individuals aged 50 and older have adenoma detection rates, increasing up to 50% by the age of 70 [32].

However, Rundle et al. conducted an analysis of screening colonoscopies in mid-risk patients aged 40–49, compared to those aged 50–59. The study revealed that both age groups had a comparable occurrence of colon adenomas: 14% in the 40–49 age range and 16% in the 50–59 age range [33]. Recently published guidelines by the American Gastroenterological Association (AGA) recommend that individuals carrying mid-risk CRC probability should commence screening protocols at age 45, while individuals having exacerbated CRC risk due to first-degree relative/s developing CRC should commence screening protocols at least 10 years prior to age of CRC development within said relative, or commence screening at age 40 [34].

The findings of this study provide valuable insights into the differences in polyp and adenoma detection rates between patients above/below 50 years of age. Elevated polyp detection rates observed in patients aged <50 years have significant implications for CRC screening and prevention strategies.

Following analysis of this investigational data, it can be seen that the majority of polyp identifications and PDRs were within the ≥50-year age group, corroborating with results presented in a recent publication by Thoma et. al. [35]. Moreover, polyps in this age group were predominantly identified within the rectum and cecum, though most sigmoid polyps were identified within the <50-year age group.

Approximately 67% of serrated lesions in this study consisted of hyperplastic polyps. However, the likelihood of such polyps developing into cancer is extremely low [36]. Colonoscopy studies typically indicate a prevalence range of 10% to 15% for hyperplastic polyps, although in specific populations, the rates can reach up to 30% [37,38]. Interestingly, when data were analyzed according to polyp localization within the colon, hyperplastic polyps in patients <50 years were predominantly detected in the rectum and sigmoid, while in the group of older patients, the detection rate of hyperplastic polyps was higher in the descendens, ascendens, and cecum colonic regions (Table A6 in Appendix A).

SSLs are typically precancerous and responsible for the development of approximately 25–33% of all CRCs [39]. Additionally, there is variability among pathologists concerning the distinguishing of hyperplastic polyps from SSLs upon detection [40]. Concerning PDR profiles for serrated polyps within this study, this was raised within the ≥50-year age group, where such polyps were predominantly found in the colon ascendens and cecum. TSAs, having villiform projections, are typically less prevalent compared to SSLs [41]. Within this study such formations were not morphologically differentiated between SSLs and TSAs, and consequently, such polyps were considered altogether, and can thus be considered as a limitation of this study.

The elimination of precancerous colon adenomas results in a 50% reduction in CRC incidence and mortality rates [42]. Within this study, there were equivalent patient populations with LGD adenomas prevalent across both age groups, though when analyzed according to colon adenoma localization, the ≥50-year age group carried the majority of LGD adenomas in the rectal region. This suggests that although LGD can be found in both age groups, there are still more such formations in patients older than 50 years. Thus, serious attention should be paid to both age groups during colonoscopy, especially to those over 50 years of age. This finding was similar for analysis of the adenoma detection rate (ADR) [20], where it was observed that ADR was elevated within the ≥50-year age group, and predominantly found in the rectum. Advanced adenomas are defined as adenomas that are either sized 10 mm or larger, exhibiting villous features, or displaying high-grade dysplasia [43]. The findings of a study presented by Kolb et.al. indicated that the prevalence of advanced neoplasia in the 45–49-year-old age group was statistically comparable to that of the 50–59-year-old population, with rates of 3.6% and 4.2%, respectively [44].

Interestingly, a higher overall polyp rate was revealed when compared to previous similar studies. This could be justified due to the meticulous attention provided by the clinical team members performing colonoscopies at our institute, with special attention to the detection and validation of individual polyps/adenomas.

Such findings were similar to our study, which demonstrated increased HGD adenoma prevalence/ADR within the ≥50 year age group, that where prevalently found within the rectum and sigmoid colon regions.

This research study did have its limitations. Firstly, the data stem from a single medical center, and are thus not fully representative of the entire CRC patient population residing within the nation. Secondly, patient ethnicity was not highly varied, which could also possibly skew evaluation inferences if not taken into consideration. In addition, confounding factors in such studies—most notably, patient gender—were recognized and adjusted for within this study as best possible, though could still have possibly affected the overall interpretation of this study’s results.

## 5. Conclusions

This investigation shed further light on the importance of age in contributing visible impact to CRC colonoscopy-based screening effectiveness, particularly concerning CRC diagnosis and management based on polyp localization evaluations. Further studies are warranted for validating such findings within larger patient cohorts. Although the majority of this study’s findings were consistent with previously established trends concerning EOCRC and LOCRC profiling, the importance of commencing CRC colonoscopy-based screening protocols below the age of 50 cannot be underestimated, due to the added value of early identification of polyps within higher-located regions of the colon in such patients, in comparison to rectum-based polyps that are typically identified in ≥50-year-old patients. Such information can undoubtedly aid CRC specialists in having increased awareness for performing better judgements in executing CRC therapeutics within such patients, thus improving survival odds. Following from this research effort, the authors aim to perform additional studies for further evaluating such findings, with larger cohorts across multiple medical centers in order to increase data robustness.

## Figures and Tables

**Table 1:** Overview of study results, focusing on cohort/s with peak prevalence of colonoscopy findings and peak prevalent colon location for such findings.

Colonoscopy Finding	Prevalent Cohort (Years Old)	Prevalent Colon Location
Overall polyp formation	≥50	Rectum
Hyperplastic polyps	<50	Rectum
Serrated polyps	≥50	Ascending colon/cecum
LGD adenomas	≥50	Rectum
HGD adenomas	≥50	Sigmoid colon/rectum

HGD—high grade diplasia.

## Data Availability

Data are contained within the article.

## Appendix A

**Table A1:** Patient profiling.

Gender n (%)	Total n = 1653	Patient Group Age		*p* between Groups <0.01
		<50 (n = 1021)	≥50 (n = 632)	
Female Male	997 (60.3%) 656 (39.7%)	609 (59.6%) 412 (40.4%)	389 (61.6%) 243 (38.4%)	<0.01 <0.01

**Table A2:** Patient populations with polyps detected during colonoscopy, according to colon regional location.

Group of Patients	Colon Region						
	Number of Polyps	Rectum	Sigmoid	Descending	Transverse	Ascending	Cecum
	n (%)	n (%)	n (%)	n (%)	n (%)	n (%)	n (%)
<50	429 (42%)	178 (41.5%)	113 (26.3%)	34 (7.9%)	58 (13.5%)	33 (7.7%)	13 (3%)
n = 1021							
≥50	483 (76%)	220 (45.5%)	117 (24.2%)	35 (7.2%)	45 (9.3%)	39 (8.1%)	27 (5.6%)
n = 632							
Total number of polyps	912 (55.2%)	398 (43.6%)	230 (25.2%)	69 (7.6%)	103 (11.3%)	72 (7.9%)	40 (4.4%)
*p*	<0.001	<0.001	<0.001	0.029	0.23	0.004	<0.001

**Table A3:** Polyp detection rate in both groups, according to colon localization.

	All Patients (n = 1653)	<50 (n = 1021)	≥50 (n = 632)
Total number of patients with polyps	732	337	395
Patients with 1 polyp	316	189	127
Patients with 2 polyps	243	74	169
Patients with 3 polyps	84	46	38
Patients with 4 polyps	64	21	43
Patients with 5 polyps	22	7	15
Patients with 8 polyps	2	0	2
Patients with 14 polyps	1	0	1

**Table A4:** Distribution of patients according to quantity of detected polyps.

Polyp dimensions	<5 mm	6–9 mm	>10 mm
Polyp quantity	462	269	181

**Table A5:** Distribution of polyps by dimension.

Patients with Hyperplastic Polyps	Total	Rectum	Sigmoid	Descending	Transverse	Ascending	Cecum
n = 234							
Patients <50 years	88	46	11	4	9	15	3
	(8.6%)	(52.3%)	(12.5%)	(4.5%)	(10.2%)	(17%)	(3.4%)
Patients ≥50 years	146	78	24	8	13	17	6
	(23.1%)	(53.4%)	(16.4%)	(5.5%)	(8.9%)	(11.6%)	(4.1%)
*p*	<0.001	<0.001	<0.001	0.042	0.043	0.08	0.078

**Table A6:** Prevalence of hyperplastic polyps in both groups.

Hyperplastic Polyps	Total	Rectum	Sigmoid	Descending	Transverse	Ascending	Cecum
n = 341							
In patients <50 years	154	82	29	7	15	18	3
	(15.1%)	(53.2%)	(18.8%)	(4.54%)	(9.7%)	(11.7%)	(1.9%)
In patients ≥50 years	187	94	32	12	18	22	9
	(29.6%)	(50.3%)	(17.1%)	(6.4%)	(9.6%)	(11.8%)	(4.8%)
*p*	<0.001	<0.001	0.02	0.025	0.051	0.027	0.009

**Table A7:** Hyperplastic polyp detection rate in both groups.

Patients with Serrated Polyps	Total	Rectum	Sigmoid	Descending	Transverse	Ascending	Cecum
n = 36							
Patients <50 years	12	0	2	2	5	1	2
	(1.2%)		(16.7%)	(16.7%)	(41.7%)	(8.3%)	(16.7%)
Patients ≥50 years	24	3	3	4	6	3	5
	(3.8%)	(12.5%)	(12.5%)	(16.7%)	(25%)	(12.5%)	(20.8%)
*p*	<0.001	0.046	0.377	0.211	0.352	0.159	0.114

**Table A8:** Prevalence of serrated polyps in both groups.

Serrated Polyps	Total	Rectum	Sigmoid	Descending	Transverse	Ascending	Cecum
n = 52							
In patients <50 years	17	0	2	3	6	3	3
	(1.7%)		(11.8%)	(17.6%)	(35.3%)	(17.6%)	(17.6%)
In patients ≥50 years	35	3	3	6	7	8	8
	(5.5%)	(8.6%)	(8.6%)	(17.1%)	(20%)	(22.9%)	(22.9%)
*p*	<0.001	0.056	0.377	0.093	0.263	0.026	0.026

**Table A9:** Serrated polyp detection rate in both groups.

Patients with LGD Adenomas	Total	Rectum	Sigmoid	Descending	Transverse	Ascending	Cecum
n = 285							
Patients <50 years	167	58	51	19	29	6	4
	(16.3%)	(34.7%)	(30.5%)	(11.3%)	(17.4%)	(3.6%)	(2.4%)
Patients ≥50 years	118	53	37	9	11	3	5
	(18.7%)	(44.9%)	(31.4%)	(7.6%)	(9.3%)	(2.5%)	(4.2%)
*p*	0.226	0.033	0.449	0.504	0.157	0.762	0.284

**Table A10:** Prevalence of LGD adenomas in both groups.

LGD Adenomas	Total	Rectum	Sigmoid	Descending	Transverse	Ascending	Cecum
n = 327							
In patients <50 years	181	62	54	22	32	7	4
	(17.7%)	(34.3%)	(29.8%)	(12.2%)	(17.7%)	(3.9%)	(2.2%)
In patients ≥50 years	146	67	43	12	14	5	5
	(23.1%)	(45.9%)	(29.5%)	(8.2%)	(9.6%)	(3.4%)	(3.4%)
*p*	0.008	<0.001	0.203	0.722	0.27	0.806	0.284

**Table A11:** LGD adenoma detection rate in both groups.

Patients with HGD Polyps	Total	Rectum	Sigmoid	Descending	Transverse	Ascending	Cecum
n = 158							
Patients <50 years	64	29	23	1	5	4	2
n = 1021	(6.3%)	(45.3%)	(35.9%)	(1.6%)	(7.8%)	(6.25%)	(3.1%)
Patients ≥50 years	94	46	33	4	6	3	2
n = 632	(14.9%)	(48.9%)	(35.1%)	(4.3%)	(6.4%)	(3.2%)	(2.1%)
*p*	<0.001	<0.001	0.001	0.054	0.264	0.785	0.628

**Table A12:** Prevalence of HGD polyps in both groups.

HGD Polyps	Total	Rectum	Sigmoid	Descending	Transverse	Ascending	Cecum
n = 173							
In patients <50 years	71	31	26	2	5	5	2
n = 1021	(6.6%)	(43.7%)	(36.6%)	(2.8%)	(7%)	(7%)	(2.8%)
In patients ≥50 years	102	50	35	5	6	3	3
n = 632	(16.1%)	(49%)	(34.35)	(4.9%)	(5.9%)	(2.9%)	(2.9%)
*p*	<0.001	<0.001	0.002	0.07	0.264	0.966	0.316
